# Development of Antiseptic and Epidermal Growth Factor Co-Loaded Thermoresponsive Composite Hydrogel for Wound Healing: Fabrication, Characterization, and In Vitro Functional Assessment

**DOI:** 10.3390/gels12060539

**Published:** 2026-06-15

**Authors:** Ting-Jui Wang, Chieh-An Chen, Yu-Hsiang Lee

**Affiliations:** 1 Interdisciplinary Program of Engineering, National Central University, Taoyuan City 320317, Taiwan; ruru.wang2783@gmail.com; 2 Department of Biomedical Sciences and Engineering, National Central University, Taoyuan City 320317, Taiwan; 112802205@cc.ncu.edu.tw; 3 Department of Chemical and Materials Engineering, National Central University, Taoyuan City 320317, Taiwan; 4 Department of Medical Research, Cathay General Hospital, Taipei City 10630, Taiwan

**Keywords:** deep wound healing, thermoresponsive, injectable hydrogel, antiseptic, growth factor, hyaluronic acid, joint therapy

## Abstract

Deep wounds often lead to severe complications such as persistent infection, biofilm formation, and high patient morbidity. While skin injuries can usually be managed with functional dressings, wounds in deep layers without sufficient treatment may serve as primary entry points for bacterial infection, thereby posing a significant life-threatening risk to patients. With the rising prevalence of chronic diseases and an aging population, effective strategies for enhanced wound healing are still in high demand. In this study, an injectable and thermoresponsive hexamethylene diisocyanate–Pluronic F127 copolymer–hyaluronic acid composite hydrogel loaded with polyhexamethylene biguanide (PHMB) and epidermal growth factor (EGF), named PEHHPG, was developed for joint therapy of deep wounds. PEHHPG self-gels at 37 °C and stabilizes both agents in the gel matrix. Based on the results of microbial colony assay and analysis of fibroblast growth kinetics, PEHHPG with ≥200 ppm of PHMB and ≥0.15 μg/mL of EGF can eradicate bacteria and enhance cell proliferation in vitro, illustrating the functionalities of PEHHPG. Given the aforementioned effects, together with the recognized advantages of injectable hydrogels such as wound shape/depth adaptation, low adhesiveness, exudate absorptiveness, and moisture maintenance, the developed PEHHPG is anticipated to be a feasible dressing material for deep wound treatment after further in vivo examinations.

## 1. Introduction

Deep wounds, often classified as complex or chronic in clinical settings, frequently complicate clinical treatment through a cycle of persistent infection, delayed healing, and substantial patient morbidity [1]. Key complications include biofilm formation, spreading of infection, tissue necrosis, and wound breakdown [2,3,4], and these complications may pose a serious risk to life. Among these complex complications is cellulitis, which is a serious, potentially life-threatening bacterial infection that requires prompt medical treatment. While many cases are managed successfully with oral antibiotics in a clinic setting, untreated or severe lesions can quickly spread to the blood, lymph nodes, or deep tissues, resulting in severe complications such as sepsis, endocarditis, or necrotizing fasciitis [5,6]. A deep wound is a major risk factor and a common entry point for bacteria that cause cellulitis [7]. With the increasing prevalence of chronic diseases and an aging population, the incidence and recurrence of wound complications (e.g., cellulitis) have risen steadily over the last decade [8], suggesting that an effective strategy for enhanced deep wound healing is still in high demand.

Currently, antibiotic therapy remains the primary treatment for infected wounds, administered either orally or locally depending on disease severity [9]. In more serious cases, particularly those involving cellulitis or abscess formation, surgical interventions such as drainage or debridement are often required. Nonetheless, several issues remain unresolved, including insufficient antibiotic levels at the site of infection, the risk of systemic side effects, and the development of antimicrobial resistance associated with repeated and/or prolonged use [10,11]. In addition, deep injury is often accompanied by delayed wound healing and incomplete tissue regeneration, which may dramatically extend treatment duration and consequently increase healthcare costs.

Localized drug administration at the injury site offers a compelling alternative to mitigate the challenges of treating deep wounds. Hydrogels serve as ideal wound dressings due to their inherent biodegradability, biocompatibility, and moisture-retention capabilities, closely mimicking the mechanical profile of the natural extracellular matrix [12,13]. Stimuli-responsive injectable hydrogels, which undergo phase transitions triggered by factors such as pH and/or temperature, are particularly valuable for medical applications [14,15]. Their fluidity allows them to adapt to irregular or deep lesions, enabling sustained drug delivery and improved healing while minimizing the risk of secondary infections or complications [16].

Pluronic F127 (PF127) is a widely recognized biocompatible, thermoresponsive triblock copolymer composed of poly(ethylene oxide) (PEO)−poly(propylene oxide) (PPO)−PEO segments. While frequently utilized in cell scaffolding and drug delivery [17,18], its clinical utility is often limited by poor mechanical stability and fast dissolution rates [19]. Consequently, reinforcing its structural integrity through chemical or physical modification is essential for practical use.

Combinational therapy through the administration of multiple wound healing agents and/or modalities is a feasible way to promote therapeutic efficacy of deep wounds since the effective dose and/or accumulation of each agent in the wound site can be simultaneously provided; therefore, the issues of bacterial resistance and insufficient tissue regeneration in the deep layer can be solved [20]. Polyhexamethylene biguanide (PHMB) is recognized for its broad-spectrum antimicrobial activity and favorable safety profile, making it a staple in clinical wound management [21,22]. Meanwhile, epidermal growth factor (EGF) is critical for modulating cellular growth and maturation, which are essential processes for wound healing [23,24]. Integrating these two agents into a unified delivery system offers a dual-action approach that simultaneously manages localized infection and promotes tissue regeneration.

In this study, we aimed to develop an injectable and thermoresponsive hexamethylene diisocyanate (HDI)–PF127 copolymer–hyaluronic acid (HA) composite hydrogel bearing PHMB and EGF, named PEHHPG, for joint therapy of deep wounds. In addition to the antibacterial and cellular proliferation effects of PHMB and EGF, respectively, the fluidic format allows PEHHPG to adequately reach wound sites in the deep layer. HDI serves as a chemical crosslinker, linking PF127 polymer chains to convert the physically associated micellar structure into a more stable covalent network, thereby enhancing the structural integrity of PF127 gels. High-molecular-weight HA (HMWHA; MW > 1000 kDa) is employed because it is an endogenous, natural polymer that is known for protecting cells from apoptosis [25,26], maintaining tissue integrity [27,28], and reducing inflammatory responses by suppressing inflammatory factor generation and blocking cell signaling [29,30]. In this study, the fabrication, characterization, and in vitro functionalities, including antibacterial effects and cell growth promotion, of PEHHPG were stepwise investigated.

## 2. Results and Discussion

### 2.1. Characterization of H-PF127 and PEHHPG

**Figure 1:**
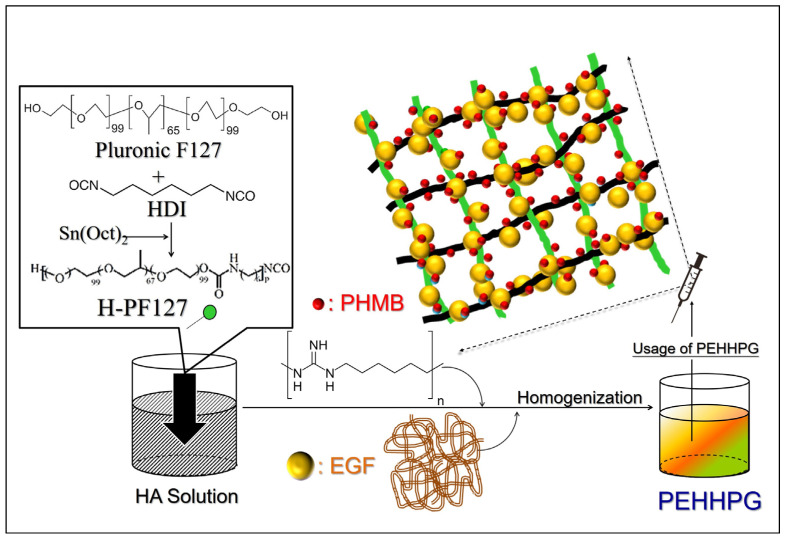
Schematic diagram showing the fabrication of PEHHPG.

**Figure 2:**
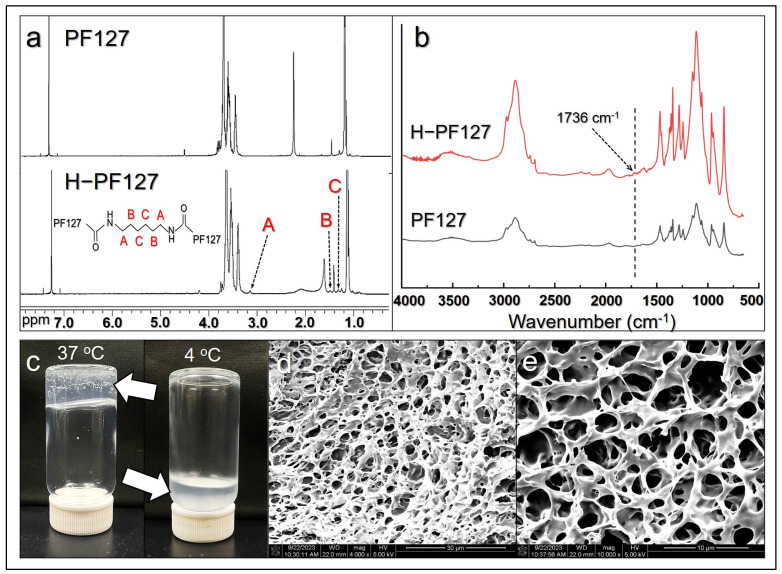
(**a**) Comparison of ^1^H NMR spectra for PF127 and the synthesized H-PF127. The peaks A, B, and C located at 3.2, 1.5, and 1.3 ppm in the H-PF127 spectrum confirm the presence of HDI aliphatic chain methylene protons in H-PF127. (**b**) FTIR spectra of PF127 and H-PF127, where the peak at 1736 cm^−1^ denotes the crosslinking of HDI and PF127. (**c**) Photographs of PEHHPG illustrating its thermoresponsive characteristics at 4 and 37 °C. (**d**,**e**) SEM micrographs showing the morphology of PEHHPG, including the surface at 4000× (**c**) and the internal architecture at 10,000× (**d**) magnification.

The preparation of PEHHPG is illustrated in Figure 1, where the developed composite hydrogel is typically stored in syringes for subsequent use. The crosslinking mechanism between HDI and PF127 was established since the nucleophilic oxygen atom from the terminal hydroxyl groups of PF127 attacks the electrophilic carbon atom of the isocyanate groups (NCO) on the HDI, forming H-PF127 with a urethane (carbamate) linkage. The gelation time of PEHHPG is approximately 40 s, while 30 min of agitation was continuously performed to thoroughly homogenize the constituents. The ^1^H NMR profiles for the precursor PF127 and its HDI-modified derivative H-PF127 are presented in Figure 2a. While PF127 and H-PF127 display characteristic signals for PPO methyl protons and PEO ethylene protons at 1.05 and 3.64 ppm, respectively, the H-PF127 spectrum uniquely exhibits additional methylene peaks at 3.2, 1.5, and 1.3 ppm (Figure 2a, points A, B, and C) that denote the features of the aliphatic chain of HDI. Similarly, the FTIR spectrum of H-PF127 exhibits a peak at 1736 cm^–1^ (Figure 2b), which corresponds to the C–O stretching vibration of the carbonyl group formed by the urethane linkage between HDI and PF127 [31], while it does not appear in PF127, indicating that the crosslinking of HDI with PF127 was successful. Overall, H-PF127 exhibits PF127 and HDI feature structures, illustrating that the association of HDI to PF127 was successfully achieved in this study.

Figure 2c shows that PEHHPG is a translucent, colorless injectable hydrogel. It demonstrates clear thermoresponsive behavior, maintaining a low-viscosity fluid state at 4 °C (Figure 2c right) while transitioning to a more viscous gel at 37 °C (Figure 2c left). This temperature sensitivity likely stems from the micellar self-assembly of PF127, where rising temperatures trigger an increase in H-PF127 micelle formation and agglomeration [32,33], allowing PEHHPG to exhibit a sol−gel phase transition in response to temperature variations. SEM imaging (Figure 2d,e) further reveals that PEHHPG has a porous network structure with a pore size of 5.16 ± 2.03 μm and a porosity of 70.5%, an architecture well suited for facilitating reagent diffusion and drug release [34].

### 2.2. Antibacterial and Cytotoxic Effects of PEHHPG

**Figure 3:**
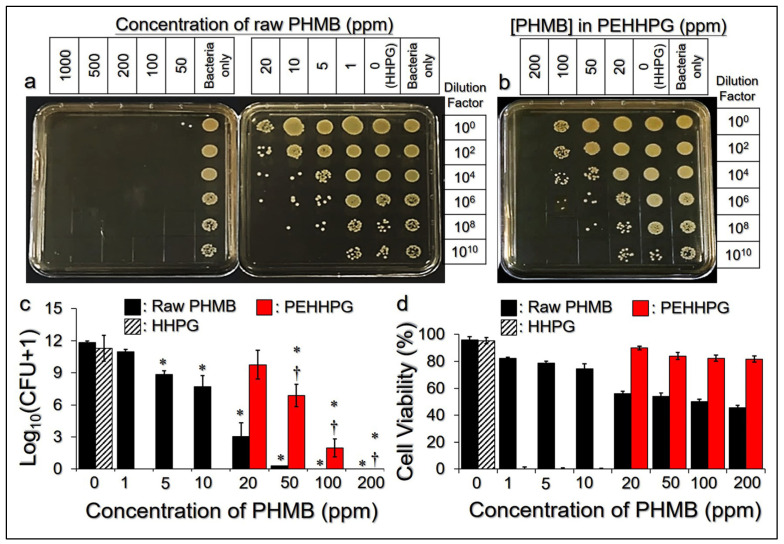
Biological effects of PEHHPG. (**a**,**b**) Photographs of *S. epidermidis* colonies after treatment with raw PHMB (**a**) or PEHHPG (**b**) containing various concentrations of PHMB as indicated in the figure. The six rows present the colony-forming conditions of the group using 10^0^–10^10^-fold-diluted *S. epidermidis* as the seeding bacteria. Both images were taken after the bacteria were plated on the TSB agar plates for 12 h. (**c**) Quantitative analyses of the BPI of *S. epidermidis* presented in (**a**,**b**). Values are presented as mean ± SD (n = 3). * *p* < 0.05 compared with the group without PHMB or hydrogel treatment. ^†^ *p* < 0.05 compared with the group with HHPG. (**d**) Cytotoxicity of raw PHMB or PEHHPG ([EGF] = 0.15 μg/mL) with various dosages of PHMB to NIH/3T3 cells. Values are presented as mean ± SD (n = 3).

The antibacterial effects of raw PHMB and PEHHPG with various PHMB dosages against *S. epidermidis* are illustrated in Figure 3a,b, respectively. Based on the BPI analysis shown in Figure 3c, 0–200 ppm free PHMB provided dose-dependent antibacterial efficacy against *S. epidermidis*. Regarding the antimicrobial capability of the hydrogel, HHPG ([PHMB] = 0 ppm) showed modest bacterial growth compared with the group without treatment, likely due to the intrinsic bacteriostatic properties of HA [35]. Notably, the association of PHMB significantly enhanced the antibacterial potency of the hydrogel. The results show that the BPI remarkably dropped by 43.6% (Figure 3c, *p* < 0.05) at 50 ppm of PHMB, and declined to zero as the dose of PHMB was elevated to ≥200 ppm, where no *S. epidermidis* colonies were observed (Figure 3a). These results clearly indicated that the antibacterial efficacy of PEHHPG is mainly contributed by the encapsulated PHMB.

The cytotoxicity of 1–200 ppm raw PHMB and PEHHPG with 20–200 ppm of PHMB toward fibroblasts was examined to assess their biological safety. According to the hemocytometric analysis presented in Figure 3d, ≥20 ppm PHMB led to <60% viability, while PEHHPG with 200 ppm of PHMB resulted in >80% of cellular viability, indicating that the toxicity of PEHHPG with ≤200 ppm of PHMB is negligible. To completely arrest microbial proliferation with minimal antibacterial agents, 200 ppm was selected as the effective dosage of the antiseptic PHMB in the subsequent PEHHPG investigations.

### 2.3. Effect of PEHHPG on Cell Proliferation

**Figure 4:**
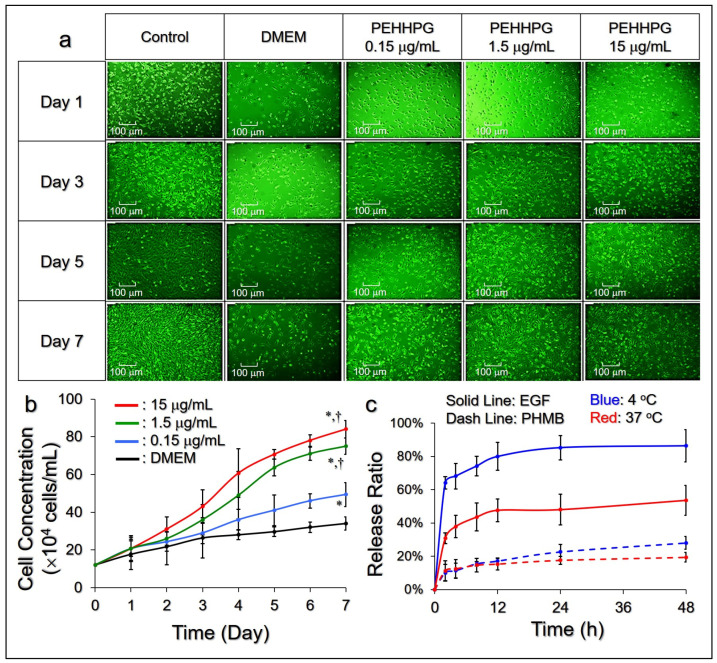
Effects of PEHHPG on cell growth and drug release kinetics of PEHHPG. (**a**) Photomicrographs presenting the growth conditions of the NIH/3T3 cells after treatment with full medium (control), DMEM, or PGCM prepared with PEHHPG containing 200 ppm of PHMB and 0.15, 1.5, or 15 μg/mL of EGF for seven days. (**b**) Growth kinetic profiles of the NIH/3T3 cells after treatment with DMEM or PGCM prepared with PEHHPG with [PHMB] = 200 ppm and [EGF] = 0.15, 1.5, or 15 μg/mL for seven days. Values are presented as mean ± SD (n = 3). * *p* < 0.05 compared with the group with DMEM. ^†^ *p* < 0.05 compared with the group with PGCM where PEHHPG contained [PHMB]/[EGF] = 200 ppm/0.15 μg/mL. (**c**) Drug release profiles of the PHMB and EGF from PEHHPG at 4 or 37 °C within 48 h. Values are presented as mean ± SD (n = 3).

Following the verification of PHMB’s antibacterial properties, we investigated how EGF influences cell growth using PEHHPG-treated conditioned media (PGCM) as a cell proliferative stimulant. Figure 4a illustrates a positive correlation between NIH/3T3 proliferation and the EGF concentration of PEHHPG applied to the PGCM preparation. Specifically, cell count analysis (Figure 4b) further revealed that PGCM enriched with 0.15, 1.5, and 15 μg/mL of EGF can accelerate the growth rates of NIH/3T3 cells by 1.4-, 1.8-, and 1.9 folds (*p* < 0.05 for each; Figure 4b), respectively, in comparison with the EGF-free control (*G*_R_ = 0.149 day^−1^). This led to a 1.45-, 2.21-, and 2.47-fold increase in total cell density over seven days. These results confirm that PEHHPG containing ≥0.15 μg/mL of EGF effectively stimulates cell growth in vitro. Consequently, 15 μg/mL of EGF was chosen for further PEHHPG evaluations to ensure sufficient/adequate dosages for promoting deep wound healing in vivo.

### 2.4. Drug Release Kinetics of PEHHPG

Figure 4c demonstrates the 48 h release profiles of PHMB and EGF loaded within the PEHHPG hydrogel at temperatures of 4 and 37 °C. A biphasic release behavior was observed in both conditions, characterized by an initial rapid delivery during the first 3 h, followed by a steady, prolonged release phase. After 48 h of the drug release process, the cumulative release levels reached approximately 28% at 4 °C and 19% at 37 °C for PHMB, while EGF showed significantly higher release of 86% at 4 °C and 53% at 37 °C. This enhanced release at the lower temperature likely stems from the phase transition of PEHHPG toward a more fluid state at 4 °C (Figure 2c), which minimizes the structural resistance within the hydrogel matrix. These findings indicate that the liberation of the loaded PHMB and EGF is highly temperature-dependent, supporting the hydrogel’s potential for wound care applications where typical skin temperatures are around 32 °C.

### 2.5. Rheology and Thermal Properties of PEHHPG

**Figure 5:**
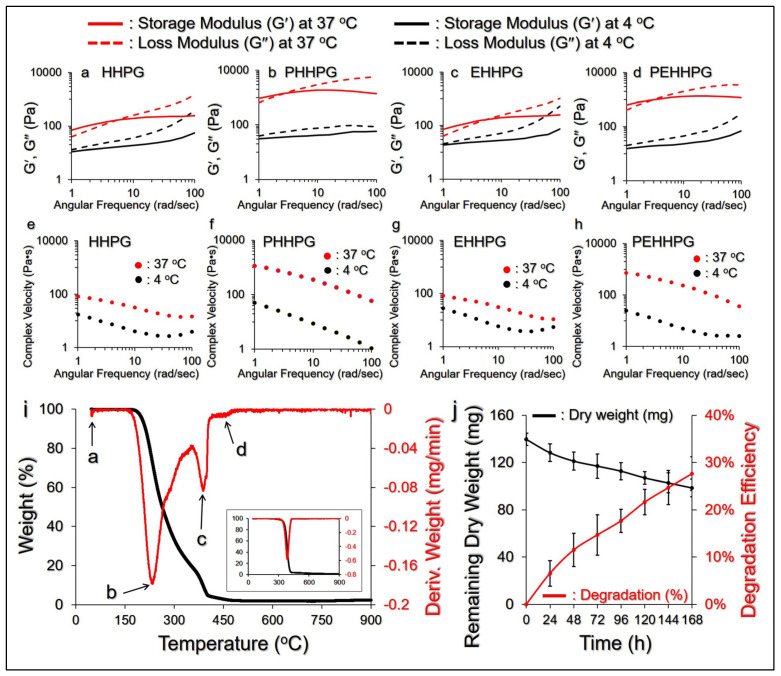
Assessment of the physicochemical properties of PEHHPG. (**a**–**h**) Curves of storage modulus (G′; **a**–**d**), loss modulus (G″; **a**–**d**), and complex viscosity (**e**–**h**) vs. angular frequency for HHPG, PHHPG, EHHPG, and PEHHPG at 4 or 37 °C. (**i**) TGA (black) and DTG (red) curves of PEHHPG heated from 50 to 900 °C with an increase rate of 10 °C/min under nitrogen atmosphere. a, b, c, and d indicate the four feature peaks in the DTG curve. The inset diagram exhibits the TGA and DTG curves of HHPG from 50 to 900 °C. (**j**) Degradation (red) and remaining dry weight (black) profiles of PEHHPG at 37 °C in PBS for 7 days. Values are presented as mean ± SD (n = 3).

The mechanical properties of HHPG, PHHPG, EHHPG, and PEHHPG with 200 ppm/15 μg/mL of PHMB/EGF under different temperatures were assessed by analyzing their rheological behaviors as a function of angular frequency. Figure 5a–h show that HHPG and its three derivatives were in a steady liquid state at 4 °C, while they shifted to the gel phase as the temperature was increased to 37 °C, indicating that neither PHMB nor EGF at the designated dosages altered the thermoresponsiveness of HHPG. In addition, PEHHPG showed increased viscosity at higher temperatures (Figure 5h), making it potentially adhesive at >30 °C. This property suggests that PEHHPG is highly suitable for use in the treatment of skin wounds in the deep layer.

The thermal properties of PEHHPG were analyzed via TGA and DTG from 50 to 900 °C, as illustrated in Figure 5i, where the DTG curve reveals four distinct thermal degradation stages as marked by points a–d. An initial 1% mass loss around 90 °C is linked to the evaporation of moisture. The primary degradation occurring between 170 and 280 °C with approximately 85% of weight reduction corresponds to the breakdown of PHMB (decomposition temperature = ~190 °C), EGF (decomposition temperature = ~250 °C), H-PF127 (*T*_b_ of HDI = 255 °C), and HA [36,37]. Subsequently, PF127 decomposes at 310–420 °C, which contributes about 12% of weight loss [38], followed by a final 2% loss near 450–500 °C due to the residual degradation of HA and PF127.

### 2.6. Stability of PEHHPG In Vitro

The degradation profile of PEHHPG was assessed by monitoring its dry weight fluctuations during incubation in PBS at 37 °C. Figure 5j illustrates that 98.5 mg of the gel remained after 7 days, corresponding to a degradation degree *D*_d_ = 27.6% post-heating. Such mass loss is likely attributed to the cleavage of ester bonds that associate the acrylate groups with the PEO-PPO-PEO copolymers, which triggers the breakdown of PF127 micelles under thermal stress as reported previously [39,40]. Based on the data presented in Figure 5j, approximately 70% of the hydrogel entity remained intact after 7 days at temperatures above 30 °C, implying that PEHHPG is particularly suitable for long-term applications in places with infrequent wound dressing replacement, such as deep wounds. Additionally, compared to the previously reported patch-format wound dressings with antibacterial and fibroblast-activating effects [41,42], PEHHPG with fluidic and thermoresponsive characteristics further offers wound shape/depth adaptation and enhanced wound retention, which are essential for treating deep wounds.

## 3. Conclusions

In conclusion, an injectable and thermoresponsive H-PF127/HA copolymer crosslinked with a composite hydrogel loaded with PHMB and EGF, named PEHHPG, was successfully developed in this study. PEHHPG self-gels at body temperature and provides antimicrobial and enhanced cell proliferation properties, as confirmed through in vitro testing. By integrating these functionalities, PEHHPG addresses common challenges in deep wound care, such as drug resistance and adverse side effects. Considering the verified effectiveness of PEHHPG along with common advantages of hydrogels used for wound healing, including wound bed adaptation, exudate absorptiveness, low adhesion (non-adherent interface), and moisture retention [43,44,45], the developed PEHHPG exhibits a high potential for deep tissue repair. While current investigations are limited to cell examinations, further in vivo evaluations are certainly required to achieve its clinical applicability, and efforts are currently in progress.

## 4. Materials and Methods

### 4.1. Synthesis of H-PF127 Copolymer

To synthesize H-PF127, 6 g of PF127 (Sigma-Aldrich, St. Louis, MO, USA) was preheated at 80 °C under vacuum for 30 min. Subsequently, 85 mg of HDI (Sigma-Aldrich) and 50 mg of stannous octoate were introduced into the molten polymer and stirred at the same temperature for an additional 30 min. After cooling to room temperature, the crude product was dissolved in 30 mL of chloroform and then precipitated into a 200 mL mixture of ethyl ether and petroleum ether (1:1 *v*/*v*). The resulting H-PF127 was isolated via suction filtration and dried under vacuum for 48 h. The final product was characterized by proton Nuclear Magnetic Resonance (^1^H NMR) and Fourier-Transform Infrared Spectroscopy (FTIR).

### 4.2. Fabrication and Characterization of PEHHPG

To fabricate PEHHPG, H-PF127 was first dissolved in deionized water (10 wt%) via vigorous stirring at 4 °C. Subsequently, HA (MW = 1200 kDa) (Sigma-Aldrich) and the desired amounts of PHMB (Sigma-Aldrich) and EGF (Sigma-Aldrich) were introduced into the H-PF127 solution in a stepwise manner, where the concentration of HA was maintained at 0.5 wt%. The mixture was continuously agitated at 1500 rpm for 30 min at 4 °C, by which PEHHPG was obtained thereafter. SEM was employed to characterize the surface and internal morphologies of the resulting composite hydrogel, in which the pore size and porosity of PEHHPG were analyzed using the software ImageJ (version 1.52a).

### 4.3. Bacterial Cultivation and Antibacterial Examination

*Staphylococcus epidermidis* (*S. epidermidis*; ATCC^®^ 12228^TM^, ATCC, Rockville, MD, USA) was cultivated aerobically in tryptic soy broth (TSB) at 37 °C, and its growth was quantitatively evaluated by spectrophotometry set at λ_abs_ = 600 nm. Upon reaching an optical density (OD_600_) of 1.0, the culture was diluted 1:100 for subsequent sub-cultivation.

The antibacterial efficacy of PEHHPG was assessed via the colony-forming assay. *S. epidermidis* with an OD_600_ = 0.2 was co-cultured with HHPG or PEHHPG (containing 0.15 μg/mL of EGF) across a range of PHMB for 18 h, followed by serial dilution and seeding on the TSB agar plates. After maintenance at 37 °C for 12 h, the antibacterial effect of each set was assessed based on the bacterial population index (BPI) calculated as the logarithm of the colony formation unit (CFU; BPI = Log_10_ ((CFU + 1)/mL)) reported previously [46]. In this study, the antibacterial activity of free PHMB at various concentrations was first evaluated, and the results were utilized for the subsequent dosage design of PEHHPG.

### 4.4. Cell Culture

NIH/3T3 murine fibroblasts (ATCC^®^ CRL-1658™, ATCC, Rockville, MD, USA) were cultured in Dulbecco’s modified Eagle’s medium (DMEM) supplemented with 10% fetal bovine serum (FBS) and 100 U/mL penicillin–streptomycin. Cultures were incubated at 37 °C in a humidified atmosphere with 5% CO_2_.

### 4.5. Evaluation of Cytotoxicity of PEHHPG

The toxicity of raw PHMB on mammalian cells was initially examined before applying it to HHPG. In brief, NIH/3T3 cells were seeded in 24-well culture plates with 2 × 10^5^ cells/well and exposed to PHMB in concentrations of 0, 1, 5, 10, 20, 50, 100, and 200 ppm. After 24 h incubation at 37 °C, the cell viability was determined via hemocytometry in association with trypan blue exclusion.

The effects of PEHHPG with 0.15 μg/mL of EGF and various concentrations of PHMB on NIH/3T3 cells were further evaluated, where the dose range of PHMB was determined based on the results of free PHMB-induced cytotoxicity described above. Briefly, NIH/3T3 cells in 24-well culture plates (2 × 10^5^ cells/well) were separately treated with PBS (blank), HHPG, and PEHHPG, in which the concentrations of intra-gel PHMB were set as 20–200 ppm. After incubation at 37 °C for 24 h, the cell viabilities were analyzed by hemocytometry in association with trypan blue exclusion.

### 4.6. Assessment of Effect of PEHHPG on Cell Growth

PEHHPG-treated conditioned media (PGCM) were prepared. In brief, PEHHPG containing the specific dose of PHMB and 0.15, 1.5, or 15 μg/mL of EGF was incubated in DMEM at 37 °C for 48 h, followed by supernatant collection and filtration. NIH/3T3 cells were seeded in 6-well culture plates with a density of 5 × 10^4^ cell per well 24 h prior to the experiment. Cell counts treated with different PGCMs were performed daily for 7 days using a hemocytometer in association with trypan blue exclusion. The growth rate (*G_R_*) of the cells for each group was calculated using the following formula:

(1) $$G_{R}\times (t_{2}-t_{1})=\ln\left(\frac{N_{t2}}{N_{t1}}\right)$$

where *N*_t1_ and *N*_t2_ denote the cell numbers obtained at time points *t*_1_ and *t*_2_, respectively.

### 4.7. Evaluation of Drug Release Kinetics of PEHHPG

To assess release kinetics, 5 mL of bubble-free PEHHPG gel containing specific doses of PHMB and EGF was placed in 20 mL of PBS. Following incubation at either 4 or 37 °C, supernatants were collected at 3, 6, 12, 24, and 48 h. The concentrations of released PHMB and EGF were then determined via spectrophotometry (λ_abs_ = 235 nm) and a human EGF ELISA kit (RayBiotech, Peachtree Corners, GA, USA), respectively, according to the manufacturer’s instructions.

### 4.8. Evaluation of Rheological and Thermal Properties of PEHHPG

The rheological properties of HHPG, PHMB-loaded HHEG (PHHPG), EGF-loaded HHPG (EHHPG), and PEHHPG with specified dosages of PHMB and/or EGF were measured using the Discovery HR-1 rheometer (TA Instruments, New Castle, DE, USA) incorporated with a temperature controller. For each sample, the storage modulus (G′), loss modulus (G″), and complex viscosity were measured as a function of angular frequency (rad/s) at 4 or 37 °C in the oscillatory mode.

The thermal properties of HHPG and PEHHPG, which contained specified doses of PHMB and EGF, were evaluated using the thermogravimetric approach (TGA, PYRIS 1, Perkin Elmer, Shelton, CT, USA) in association with derivative thermogravimetric (DTG) analysis after sample lyophilization. Both freeze-dried hydrogel specimens were heated from 50 to 900 °C at a constant temperature with an increase rate of 10 °C/min under nitrogen flow.

### 4.9. Analysis of Degradation of PEHHPG In Vitro

A 7 mL volume of PEHHPG containing specified doses of PHMB and EGF was split equally into seven tubes. Each PEHHPG sample was immersed in PBS (PEHHPG:PBS = 1:2 (*v*/*v*)) and incubated at 37 °C in the dark. One tube of PEHHPG specimen was lyophilized and weighed every 24 h for 7 days. The degradation efficiency (*D_E_*) of PEHHPG was assessed by using the following formula:

(2) $$D_{E}=\frac{W_{0}-W(t)}{W_{0}}\times 100\%$$

where *W*_0_ represents the original dry weight of PEHHPG before immersion in PBS, while *W*(*t*) denotes the dry weight of the hydrogel measured at a specific time *t* > 0 after lyophilization.

### 4.10. Statistical Analysis

All data were presented as mean ± standard deviation (SD) with n ≥ 3. Statistical analysis was performed using the software MedCalc (version 17.2), in which multiple comparisons were analyzed with two-way ANOVA followed by Dunnett’s post hoc test. The significance level was defined as *p* < 0.05 based on Student’s *t*-test throughout this study.

## Data Availability

The data presented in this study are available upon request from the corresponding author.
